# Deep sea as a source of novel-anticancer drugs: update on discovery and preclinical/clinical evaluation in a systems medicine perspective

**DOI:** 10.17179/excli2014-632

**Published:** 2015-02-10

**Authors:** Patrizia Russo, Alessandra Del Bufalo, Massimo Fini

**Affiliations:** 1 Laboratory of Molecular Epidemiology, IRCCS "San Raffaele Pisana", Via di Val Cannuta, 247-249, Rome, Italy; 2 Scientific Direction IRCCS "San Raffaele Pisana"; Via di Val Cannuta, 247-249, Rome, Italy

**Keywords:** deep-sea-derived drugs, cancer, systems medicine, network, therapy, preclinical studies, clinical studies

## Abstract

The deep-sea habitat is a source of very potent marine-derived agents that may inhibit the growth of human cancer cells “*in vitro*” and “*in vivo*”. Salinosporamide-A, Marizomib, by *Salinispora species* is a proteasome inhibitor with promising anticancer activity (Phase I/II trials). Different deep-sea-derived drugs are under preclinical evaluation. Cancer is a complex disease that may be represented by network medicine. A simple consequence is the change of the concept of target entity from a single protein to a whole molecular pathway and or cellular network. Deep-sea-derived drugs fit well to this new concept.

## Introduction

“*Consider the subtleness of the sea; how its most dreaded creatures glide under water, unapparent for the most part, and treacherously hidden beneath the loveliest tints of azure. Consider also the devilish brilliance and beauty of many of its most remorseless tribes, as the dainty embellished shape of many species of sharks. Consider, once more, the universal cannibalism of the sea; all whose creatures prey upon each other, carrying on eternal war since the world began.”*

*Herman Melville, Moby Dick*.

The ocean explorations start since 4500 BC when people of Mesopotamia, Greece and China began to dive into the sea to collect food, make commerce (i.e. pearl-diving industry) and possibly war [http://www.seagrant.wisc.edu/madisonjason11/timeline/index_4500BC.html]. The first testimony of marine medicine comes from 2953 BC during emperor Fu His in China as a tax for profits of fish-derived medicine (Jia et al., 2004[18]). A Greeks epigraphy at the Sanctuary of “Asklepios” at Epidaurus (4^th^ century BC) refers to a missed sponge fishermen (Guarducci, 1995[11]). Thus, on 400 BC Hippocrates reported that sea sponges has antibiotic effects and recommended to put them on soldiers' wounds (Riddle, 1987[35]). Now, it has been shown that sponges produce bioactive secondary metabolites (SMs), that origin directly by sponges or indirectly by their associated microorganisms, that could work to fight infections (Laport et al., 2009[21]). Currently, technological innovations in underwater vehicles such as submersibles, ROVs (Remotely Operated Vehicles), and AUVs (Autonomous Underwater Vehicles) allow the exploration of the depths of the ocean and in turn allow the discovery of novel marine species potentially useful in medicine. The “ocean” [from Ancient Greek Ὠκεανός (Okeanos)] occupies two-thirds of planet's surface. It is divided in 4 ocean zones (from shoreline to open ocean): *(1)* the *Intertidal Zone*, *(2) *the *Neritic Zone*, *(3) *the *Oceanic Zone*, and *(4) *the *Benthic Zone* that underlies all the other zones. Technically, the deep sea is located below a depth of 200 meters, further divided into deep sea zones. As a consequence of thermal or pressure interfaces each zone has characteristic life and conditions. The deep-water ecologist Koslow defines the deep sea “*as the area below which photosynthesis can function*” (Koslow, 2007[20]). Two zones are biologically interesting such as: *(1)* the mesopelagic zone, the “Twilight Zone” (between 200 meters to 700 or 1000 meters down), where, depending on water clarity (transparency or clearness), photosynthesis can take place; and *(2)* the boundary between the mesopelagic and the bathypelagic zone that contains the deep scattering layer - a layer of fish, squid, crustaceans etc, that migrate each day from the deep ocean to the shallows at night (Koslow, 2007[20]). In this review, we exemplify current deep sea (below 200 meters equivalent to 656 feet) drug discovery, and development of products as anticancer agents with specific examples. Drugs derived by organisms that live upper 200 meters, such as *Aplidin*^TM^ (PharmaMar USA Inc.) by *Aplidium albicans *or *Yondelis*^TM ^(Formerly ET-743, PharmaMar in partnership with Johnson & Johnson Pharmaceutical Research & Development, L.C.C. -J&JPRD-) by *Ecteinascidia turbinate* are not discussed here, since the producing organisms live in shallow waters (Koslow, 2007[20]). *Aplidium*, as well as *Ecteinascidia,* belong to tunicates family, tunicates of the genera *Aplidium* can habit in different area of the ocean including area under 200 meters and in cold water (Núñez-Pons et al., 2012[31]).

This review likes not to be a merely clinical comprehensive “list” of updated preclinical/anticancer marine drugs (successful or not), but would like to highlight the complexities of discovering and of utilizing deep sea-derived compounds into clinical trials. A great success is Ziconotide (*Prialt*^TM^ Eisai Ltd.), a synthetic form of peptide extracted from the venom of predatory tropical cone snails *Conus magus*, living under 2000 meters in the sea (Lewis et al., 2012[22]). Prialt^TM^ was licensed by EMA (European Medicine Agency] as anti-pain drug [*Doc. Rif. EMEA/772122/2009 EMEA/H/C/551; *http://www.ema.europa.eu/ema/index.jsp?curl=pages/medicines/human/medicines/000551/human_med_000989.jsp&mid=WC0b01ac058001d124]. 

## Actinomycetes

*Actinomycetes* are a subgroup of the *actinobacteria*, Gram positive bacteria with a high G+C ratio in their DNA. *Actinomycetes* are a prolific source of secondary metabolites (SMs) that account for more than 50 % of antibiotics discovered to date (Manivasagan et al., 2013[25]). Marine-derived *actinomycetes* are a “focus” in the search of novel SMs because of their diversity and their established capability to produce molecules of pharmaceutical importance (i.e. actinomicyn) (Manivasagan et al., 2013[25]). The description of the natural role of SMs is beyond the objective of this review; however it is important to remind that SMs are produced by large gene joints, including those encoding tailoring enzymes and mechanisms of resistance and transport (Jensen, 2010[17]). Organisms produce SMs since they play important ecological roles such as nutrient acquisition, chemical communication, and defense (Jensen, 2010[17]).

The genus *Salinispora *is the first obligate seawater-requiring marine *actinomycete* (Mincer et al., 2005[29]). This genus is widely distributed in tropical and subtropical ocean sediments with the species *Salinispora* including *S. tropica, S. arenicola*, and *S. Pacifica *(Mincer et al., 2005[29]).* Salinispora tropica* is found in tropical and subtropical marine sediments around the world at the depth of up to 1100 m (Mincer et al., 2005[29]), is an aerobic, Gram-positive, non-acid-fast *actinomycetes* that form extensively branched substrate hyphae carrying smooth-surfaced spores singly or in clusters. *S. tropica *strain CNB-440 has a single circular chromosome composed of 5,183,331 bp, with no plasmids, and an average G-C content of 69.5 % (Udwary et al., 2007[40]). *S. tropica *produces the potent proteasome inhibitor Salinosporamide-A.

## Salinosporamide-A

Salinosporamide-A [C_15_H_20_ClNO_4_ IUPAC name: (4R,5S)-4-(2-chloroethyl)-1-((1S)-cyclohex-2-enyl(hydroxy)methyl)-5-methyl-6-oxa-2-azabicyclo[3.2.0]heptane-3,7-dione) or NPI-0052- or Marizomib] belongs to the family of salinosporamides, characterized by a densely functionalized γ-lactam-β-lactone bicyclic *core*, that is responsible for its irreversible binding to the β subunit of the 20S proteasome. The ubiquitin-proteasome-system (UPS) is a cellular protease responsible for intracellular nonlysosomal proteolysis of aberrant folded or short-living proteins, complementing the function of lysosomes. Thus, UPS is involved in signal transduction, cell cycle regulation, cell differentiation and apoptosis (Groll and Potts, 2011[10]). In eukaryotes, the 26S proteasome comprises a 20S proteasome or *core* particle (CP) capped by either one or two 19S regulatory particles (RPs). The 20S proteasome core particle (CP) is the proteolytically active key https://www.google.it/search?safe=active&biw=1024&bih=605&tbm=isch&sa=1&q=proteasome+26s&oq=+proteasom (Marguerat et al., 2012[26]; Kish-Trier et al., 2012[19]). Proteasomes may cleave specific amino acid residues and, according to these activities, they are classified as: *(a)* chymotrypsin-like, *(b) *trypsin-like and *(c) *caspase-like and are associated with the β5, β2, and β1 subunits, respectively. Dysfunction of UPS components is implicated in the occurrence of many human pathological disorders, including cancers, cardiovascular diseases, viral diseases, and neurodegenerative disorders (Bedford et al., 2011[3]). Pharmacological inhibition of UPS was originally considered lethal for all cell types, however the observation that tumor cells are more sensitive to proteasome inhibition than normal cells points to proteasome as a novel target for cancer treatment. Bortezomib (PS-341; marketed as *Velcade* by Millennium Pharmaceuticals or *Cytomib* by Venus Remedies) is the first proteasome inhibitor approved for the treatment of chemorefractory multiple myeloma (Cao et al., 2013[5]).

Unlike to others proteasome inhibitors such as Bortezomib and Carfilzomib (marketed under the trade name *Kyprolis* by Onyx Pharmaceuticals, Inc.) that are selective for the chymotrypsin-like activity of the proteasome, Salinosporamide inhibits all three enzymatic activities of the proteasome with preferential activity *versus* chymotrypsin and trypsin (Chauhan et al., 2005[6]) but also activates caspase-8 and 9 (Ruschak et al., 2011[36]). The antineoplastic activity of Salinosporamide is primarily on caspase-8-mediated signaling pathways so that it may induce apoptosis in cells resistant to Bortezomib. Salinosporamide A shows low cytotoxicity *versus* normal lymphocytes and bone marrow derived stem cells (Chauhan et al., 2005[6]). Salinosporamide (NPI-0052, marketed as *Marizomib* by Nereus Pharmaceuticals) is orally bioactive, inhibits tumor growth in mice, is well tolerated, and prolongs survival. The mechanisms of action and preclinical activity of Salinosporamide is reviewed intensely in literature (Potts et al., 2011[33]; Buac et al., 2013[4]; Allegra et al., 2014[2]). This compound displays potent and highly selective activity in The National Cancer Institute's (NCI) 60-cell-line panel with a mean GI_50_ value (the concentration required to achieve 50 % growth inhibition) of less than 10 nM and a greater than 4 log LC_50_ differential between resistant and susceptible cell lines. The greatest potency was observed against NCI-H226 (considered inconclusive for p53 status) non-small cell lung cancer, SF-539 CNS (inconclusive for p53 status) cancer, SK-MEL-28 melanoma (p53 mutation L145R), and MDA-MB-435 breast cancer (all with LC_50_ values less than 10 nM). NCI's-60 cell line panel is the most extensively characterized set of cells in existence frequently used as a screening tool for drug discovery [http://dtp.nci.nih.gov/branches/ btb/ivclsp.html]. In human clinical preliminary study *Marizomib* resulted generally well tolerated at effective doses. Common AEs (adverse events) include fatigue, nausea, vomiting, headache, dizziness, and fever. Dose limiting toxicities (DLT) include transient hallucinations, reversible cognitive changes; however peripheral neuropathy, thrombocytopenia and neutropenia were not seen. Twice weekly dosing with longer infusions is active with manageable toxicity (Richardson et al., 2011[34]). Table 1(Tab. 1) shows the current status of clinical studies of *Marizomib.*

The EMA Committee for Orphan Medicinal Products in June 2014 adopted a positive opinion recommending Triphase Accelerator's (a private drug development company) *Marizomib* as an orphan medicinal product for the treatment of multiple myeloma in the European Union (EU) [http://regulatory affairs.pharmaceutical-business-review.com/ news/ema-committee-recommends-orphan-drug-status-to-triphases-myeloma-drug-marizomib-240614-4301119]. The EMA decision follows the US Food and Drug Administration (FDA) granting of Orphan Drug Status to Marizomib in March 2014 [http://www.orphan-drugs.org/tag/Marizomib/#sthash.OJtfb9BH.dpbs].

## Deep Sea Derived Drugs in Preclinical Study

Table 2(Tab. 2) shows the current status (last five years) of preclinical studies of deep-sea-derived drugs potentially useful in anticancer therapy. (References in Table 2: **Sorbicillamines A-E (1-5):** Guo et al., 2013[12]. **Pyrazin-2(1H) as lead for the development of ATP competitive PK inhibitors:** Pinchuk et al., 2013[32]. **Anthranilic acid derivatives, penipacids A-E(1-5):** Li et al., 2013[23]. **Sungsanpin (1), 15-amino-acid peptide:** Um et al., 2013[41]. **Marthiapeptide A, tristhiazole-thiazoline-containing cyclic peptide:** Zhou et al., 2012[45]. **Diketopiperazines:** Zhang et al., 2013[43]. **SD118-xanthocillin X (1):** Zhao et al., 2012[44]. **Bis (2-ethylhexyl) phthalate (BEHP):** Moushumi Priya and Jayachandran, 2012[30].)

## Cancer Complexity : How Systems Biology Embracing Systems Medicine May Help to Develop Better Anticancer Therapeutic Strategies

**Figure 1:**
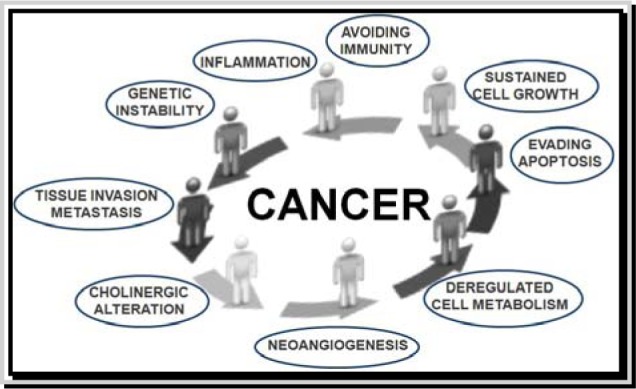
Hallmarks of cancer. This illustration shows different hallmark capabilities that were originally proposed in 2000 by Hanahan and Weinberg and then modified in 2010 by the same authors. Figure is adapted by http://www.istockphoto.com/vector/cycle-stickman-2-0-10708124?st=0e7e21f

Cancer is a complex disease representable in the form of a high nonlinear system characterized by continuous changes in different signaling pathways and molecular networks regulated by complex behaviors such as self-organization, dissipative structures, biological clock, fractals and chaos. Hundreds to thousands genetic and epigenetic alterations occur inside a cancer cell resulting in expression of aberrant or abnormal proteins that may lead to uncontrolled transcriptional and translational regulation. Complex interactions with microenvironment are also involved (Du and Elemento, 2014[8]; Li and Mansmann, 2014[24]; Masoudi-Nejad and Wang, 2015[27]; Werner et al., 2014[42]; Tang and Aittokallio, 2014[39]). Scientific progress allowed the identification of various hallmarks underlying the cancer phenotype (Figure 1(Fig. 1)) (Hanahan and Weinberg, 2000[14], 2011[13]), although more hallmarks remain to be elucidated. 

It is well known that “complex systems relay on the use of mathematical tools based on networks or graphs, where the individual parts translate to nodes and the interactions translate to edges or links” (Saetzler et al., 2011[37]). A biological network, that is considered scale-free, may be mathematically represented as a matrix or graphically as a network diagram composed by graph points or nodes, that represent genes or proteins, connected by lines representing interactions (edges). As a result, the centrality of a node may be easily measured using computational methods (Saetzler et al., 2011[37]) and the strength or confidence of the edge may be weighted. The “constituents” of the structure may be simple as proteins (nodes) or protein-protein interactions (as edges) or complicated as drugs and target proteins (differently shaped nodes) or drug-target bindings (edges) (Chen et al., 2013[7]). According to the principle of “network medicine” each signaling network is an extremely dynamic entity. Dynamic cellular networks have a finite number of states that may be represented as landscapes. This dynamic nature is the critical property of cancer signaling network that may be perturbed by mutations, that, at a systems-level, ultimately may impair the normal cell physiology and allow cells to acquire key cancer hallmarks such as uncontrolled/sustained cell-proliferation and apoptosis escape (Hanahan and Weinberg, 2000[14], 2011[13]). Currently, the knowledge of these molecular interaction networks is a key challenge to achieve a deeper understanding of cancer biology and to identify novel drug targets. The knowledge of specific biochemical pathways, as well as the knowledge of their environmental modifications (i.e. oxidized posttranslational modifications of proteins), integrated by several “omics” data (i.e. genomics, transcriptomics, proteomics, metabolomics) may have great power to identify relevant interactions that in turn may allow to determine drug targets (or combination of) yielding the optimal benefit with minimal adverse consequences. A simple consequence of network perspective is the change of the concept of target entity from a single protein to a whole molecular pathway and/or cellular network. Salinosporamide A is an inhibitor of UPS (Einsele, 2014[9]). Proteasome, constituting up to 5 % of the total protein content, represents the second-most abundant protein complexes (Marguerat et al., 2012[26]). It is a collection of complexes based on the 20S proteasome core particle (20S CP). 20S is a complex formed by 28 subunits housing proteolytic sites inside its hollow interior (Kish-Trier et al., 2012[19]). Proteasomes are found in *archaea*, some eubacteria, and in eukaryotes. Proteasome is considered as one of the most important cellular protein degradation machinery. It functions as a “gatekeeper” controlling the execution of protein degradation thus playing a crucial role in the ubiquitin-proteasome pathway (UPP). Proteasome works catalyzing the hydrolysis of amide bonds adjacent to a variety of amino acids through chymotrypsin-like, trypsin-like and post-glutamyl peptide hydrolyzing activity. UPS degrades around 80-90 % of the total intracellular proteins aberrantly folded or typically short-lived whereas the remaining 10-20 % is degraded by the lysosomes (Settembre and Ballabio, 2014[38]). UPS is a promising significant therapeutic target since it is involved in many cellular processes and pathways, such as proteins turnover, quality control of proteins, transcription, cell signaling, cell cycle, apoptosis, inflammation, immune response including antigen presentation, and development. Indeed, malfunction of UPS components (i.e. lack of proper control over UPS) is involved in different human diseases and is attributed to both cancer and to neurodegenerative disorders, yet in different context and direction (Jara et al., 2013[16]). The UPS regulates the turnover of p53 and p27^Kip1 ^as well as the activation of nuclear factor kappa B (NF-κB) signaling, thereby supporting cancer cell survival and proliferation (Jara et al., 2013[16]). Salinosporamide A potentiates the apoptosis induced by tumor necrosis factor alpha (TNF), Bortezomib, and thalidomide down-regulating the gene products that mediate cell proliferation such as cyclin D1, cyclooxygenase-2 (COX-2), and c-Myc, cell survival factors such as Bcl-2, Bcl-xL, cFLIP, TRAF1, IAP1, IAP2, and survivin, invasion such as matrix metalloproteinase-9 (MMP-9) and ICAM-1, as well as angiogenesis (vascular endothelial growth factor [VEGF]). Salinosporamide A also suppressed TNF-induced tumor cell invasion and receptor activator of NF-κB ligand (RANKL)-induced osteoclastogenesis and suppresses both constitutive and inducible NF-κB activation (Anh et al., 2007[1]).

## Conclusions

The majority of “targeted-drugs” used now in cancer therapy has the limitation to affect a single gene or a single protein, or in some cases multiple kinases. This “reductionist” approach may lead to wrong decisions as shown, for an example, by Janes et al. (2005[15]) who found that, according on the state of the signaling network, the protein Jun N-terminal kinase may have either a pro- or an antiapoptotic effect. The “integrated network modeling” suggests that a drug candidate shall take into account the ability of the system itself to adapt to perturbation (e.g., drug resistance) proposing a new approach called “magic shotguns” to find a few special molecule that broadly disrupts the whole diseases process. Deep-sea-derived compounds, as exemplified by Salinosporamide, fit well into this concept.

## Figures and Tables

**Table 1:**
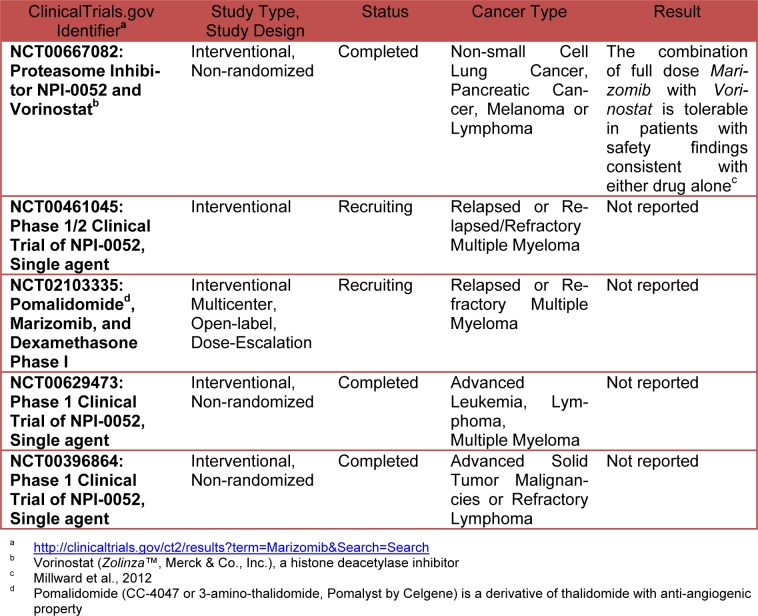
Current status of clinical studies of Marizomib

**Table 2:**
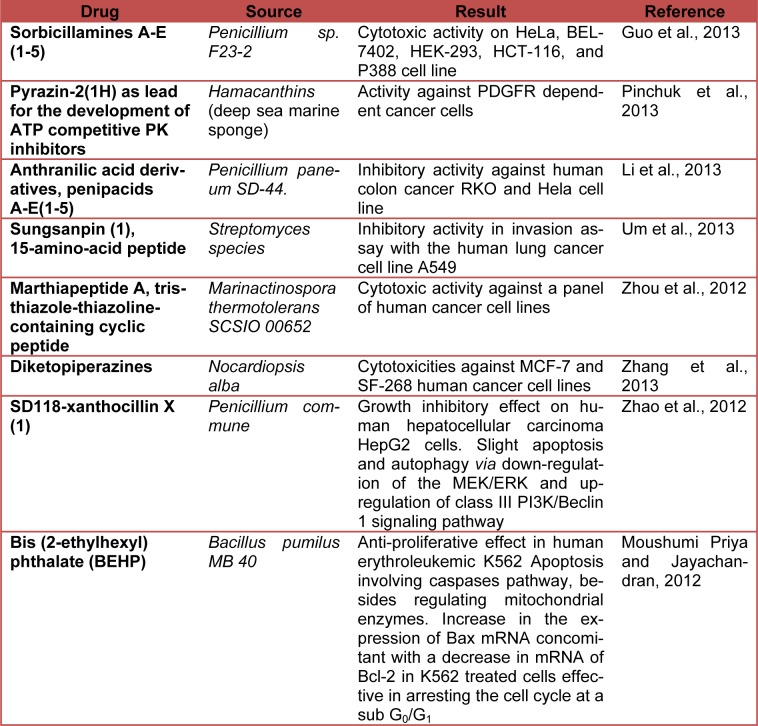
Preclinical studies of deep-sea-derived drugs potentially useful in anticancer therapy
